# Wear Behavior of Fiber Laser Textured TiN Coatings in a Heavy Loaded Sliding Regime

**DOI:** 10.3390/ma5112360

**Published:** 2012-11-16

**Authors:** Laura Vandoni, Ali Gökhan Demir, Barbara Previtali, Nora Lecis, Daniele Ugues

**Affiliations:** 1 Department of Applied Science and Technologies, Politecnico di Torino, Cso Duca degli Abruzzi, 24, Torino 10129, Italy; E-Mail: laura.vandoni@polito.it; 2 Department of Mechanics, Politecnico di Milano,Via La Masa, 1, Milano 20156, Italy; E-Mails: ali.demir@mail.polimi.it (A.G.D.); barbara.previtali@polimi.it (B.P.); nora.lecis@polito.it (N.L.)

**Keywords:** laser surface texturing, active fiber laser, TiN coating, sliding wear, boundary lubrication regime, adhesive wear

## Abstract

In heavy loaded mating components, such as sliders and sliding bearings, guaranteeing the efficiency of lubricant films for long times during severe service conditions is very complicated. In this work, the benefits deriving from the use of fiber laser sources for surface texturing of very thin TiN coatings in severe wear working conditions were demonstrated. Evaluations of the laser textured dimples shape, geometry and density are given. Wear performance of the fiber laser textured surfaces was evaluated in discontinuous oil lubricated conditions with a flat contact. High normal load and low sliding speed were applied. Comparison tests were also performed on commercial TiN and WC/C coatings. In terms of average wear volume and maximum wear depth, Laser Surface Texturing of TiN provided respectively a 70% and a 45% reduction if compared to plain TiN. If compared to WC/C the wear resistance gains were lower but LST TiN maintained such benefits for longer wear runs. SEM analysis also revealed that the laser interaction provided a localized thermal cracking to the TiN coating. However, the sliding action caused very limited and localized coating fragmentation or delamination.

## 1. Introduction

As environmental restrictions on the use of lubricants become tighter and costs associated with their disposal increase, there is a growing demand for low friction materials that allow mating surfaces to slide one against each other with reduced friction and wear [1]. This is particularly critical in load bearing components such as sliders and sliding bearings, which are prone to severe adhesive wear, as they are commonly subjected to heavy normal loads and to low-to-medium sliding speed. Surface modifications through coatings or surface texturing are viable methods for enhancing tribological properties of mechanical components. Amongst different processes used, laser surface texturing (LST) is an established method, which is utilized to generate micro pockets, commonly in form of micro dimples, on the surface of the mechanical component that mainly act as lubricant reservoirs during the sliding contact [2]. In addition, they can also entrap the worn particle and debris to reduce wear provided by third body effect and locally generate micro-hydrodynamic pressure distributions, which in turns enhance the load support capacity and increase the film thickness [3,4,5,6]. The overall effect is the reduction of friction and wear, resulting in lower energy consumption and increased lifetime of the components. Apart from tribological applications, wetting [7] and optical [8] properties of the textured surfaces, nanostructure formations inside dimples formed by ablation [9] are other points of interest in laser surface texturing research. Compared to other methods to texture surfaces, the laser process provides high flexibility of the obtainable dimple geometries with elevated precision and excellent control of the shape and size. Furthermore, it allows short preliminary preparation and post processing, as it does not require adapted dies or tools and it is environmental friendly.

Laser texturing has been applied to various tribological systems based on metallic materials, including the ring/liner assembly of engines, mechanical seals and bearings. LST has been shown to reduce the friction coefficient by 30% in piston ring applications [10,11,12]. A similar positive effect of laser surface texturing on frictional performance of face seal was also demonstrated [13]. Under the boundary lubrication regime, LST was observed to expand the range of the hydrodynamic lubrication regime in terms of load and sliding speed for both high- and low-viscosity oil lubricants [14]. Promising results have been also reported in the field of WC/Co turning cutting tools recently [15]. Besides the traditional applications on metallic materials, the use of LST was also expanded to ceramic surfaces where tribological behavior was also improved compared to un-textured polished surfaces [16]. From the point of view of the used laser sources, several types differing in wavelength and pulse width have been used for surface laser texturing. In particular, CO_2_, Nd:YAG, excimer lasers, operating in fundamental wavelengths or other harmonics, with pulse widths ranging from microsecond to nanosecond, have been employed [17,18,19,20]. More recently, the research in the field has been guided towards new trends of reducing the dimension of textured features, application of solid lubricant inserted into laser formed micro-reservoirs and moreover to the use of ultra fast lasers operating with ps and fs pulses. For this purpose, it is known that due to their relatively shorter pulses, ps and fs lasers reduce thermal damage compared to nanosecond lasers [21]. Furthermore, femtosecond laser ablation has been studied for the crater formation and hydrostatic properties of single craters [22]. Applications of Laser Surface Texturing on thin coatings are also reported [23,24,25,26,27]. This is particularly interesting for the potential creation of hard surfaces, which can be also effectively lubricated. Actually, many of the hard and wear resistant thin coatings available at research or commercial levels offer medium value of friction coefficient and sometime suffer by adhesive wear in variable loaded conditions [28,29,30,31]. To improve this aspect, for instance, it was reported that the wear life of burnished MoS_2_ film in laser textured nickel-based composite surfaces was found to be significantly higher than that for the same film on plain surfaces [32,33]. Laser surface textured TiCN coatings with 10–20 µm dimples were also reported as filled with solid lubricants using burnishing and sputtering, such practices being capable of highly improving the wear properties compared to the monolithic TiCN [34]. In such studies the tribological properties of LST coatings were always studied in point contact configuration. Despite extensive research, laser surface texturing has not been widely applied in industry. As a process that consists of generating some thousands of micro craters on relatively large surfaces, LST requires a stable and robust production tool, providing high productivity, flexibility, reduced capital and maintenance costs so as to meet the economy of scale required by industrial use. From this point of view, pulsed fiber laser systems operating with nanosecond pulses are a promising new option for LST, as they can provide high productivity along with sufficient quality. Fiber lasers are characterized by high brilliance and high focusability, resulting in beams of high quality and very small diameter; high pulse energies and repetition rates allow higher productivity. Due to their simplicity, robustness and reduced cost, they are appealing for industrial applications. The application of pulsed fiber lasers to LST is a very recent subject, being rarely reported on metallic materials [35] and on ceramic coatings [36].

In this work, laser surface texturing on TiN coating is applied with an active fiber laser system. In particular, the main aspects of fiber laser surface texturing studied are: the tight control of thermal alterations and depth of machining. The main focus is to investigate whether such characteristics may be obtained with laser sources using long pulses, which are less accurate than those using ps and fs ones, but are in turns very cost effective and highly productive. Topography and nanohardness properties of the as deposited coatings are reported. An assessment of single dimple morphology and dimples density is given. Evidence of thermal cracks within the laser affected zones (the so generated dimples) is also provided. One of the challenges of this work is to demonstrate that even if such thermal cracks are locally introduced in the coating, the textured coated surface is able to withstand severe wear conditions. To this purpose, the effect on wear resistance of a specific dimples pattern laser textured on TiN was evaluated in comparison with WC/C and plain TiN coatings. The wear test was carried out in discontinuous oil lubricated conditions. High load and low velocity sliding conditions were investigated using two flat sliding surfaces, simulating wear conditions of plane sliders. Recorded values of friction coefficient, average wear volume and maximum wear depth for the three coating grades are given, together with a detailed study of wear tracks morphologies. Marked improvement in wear resistance were achieved through laser surface texturing of TiN with respect to the other two studied coatings. A description of wear mechanisms acting on the different coating grades and a discussion of the reasons for the improvements achieved through surface texturing are finally reported.

## 2. Experimental Section

### 2.1. Sample Preparation and Their Quality Assessment

A set of oil quenched and tempered 42CrMo4 steel disks of 50 mm diameter were used as substrate materials. Such steel is a general purpose engineering steel frequently used in load bearing components such as bearings and gears. The heat treatment applied on substrate includes: austenitising at 850 °C—quenching in oil—tempering at 600 °C to achieve a hardness of 32HRC. The samples were first gross machined, ground and polished with an automatic polishing machine using both abrasive papers and diamond paste impregnated discs. On such samples two commercial monolayer coatings were deposited with industrial Physical Vapor Deposition (PVD) equipment: a self-lubricant WC/C coating deposited by sputtering process and a hard TiN coating deposited by cathodic arc process. A preliminary measurement of the coating thickness through the ball on crater test was performed. Scanning Electron Microscopy (SEM) analysis using LEO 1450 VP was used to investigate the surface morphology of coatings and especially the presence of defects in the as deposited layer. Nanohardness and reduced modulus were evaluated using a nanoindenter Hysitron UBI 950 with a Berkovich tip and applying 10,000 µN with a trapezoid load history at constant loading rate.

### 2.2. Laser Surface Texturing

Laser surface texturing process was designed to obtain low aspect ratio dimples with controlled depth and without dross. Texturing was applied only on the hard TiN coating. In the case of laser surface texturing of thin layers, the control of dimple depth is evidently important to avoid reaching the substrate. Having in mind such aspects, the texturing pattern was conceived to be formed by craters with diameter *ca.* 50 µm and depth less than 2 µm, which is very close to the thickness of the coating as reported below. An IPG Photonics Q-switched fiber laser was used. Maximum average output power of the used laser is 50 Watts and generates pulse repetition rates between 20 and 80 kHz and pulse width in the order of nanoseconds (about 100 ns FWHM). The laser power is variable with pump current percentage between 10% and 100%.The collimated beam exiting the laser system has a diameter of 5.9 mm, which is then focused with a 60 mm focal lens inside a Laser Mech micromachining head. Theoretical value of the beam waist diameter in this combination is 23 µm. However, the laser beam was focused at 0.38 mm below the material surface to have a larger spot on the surface, which had a calculated value of 44 µm. The system specifications are reported in Table 1.

**Table 1:** Specifications of the laser system.

Laser System Parameters	Numerical Values
Maximum average power	50 W
Wavelength	1064 nm
Pulse repetition rate (PRR)	20–80 kHz
M^2^	1.7
Collimated beam diameter	5.9 mm
Focal length	60 mm
Beam waist diameter	23 µm

A ramped pulse train was used for the machining of single dimples. The ramped pulse train was generated with 65 µs modulation duration with 100% pumping current at 50 kHz of pulse repetition rate. The estimated energies of the pulses are reported in Table 2. For material positioning two Aerotech ALS-1350 linear stages (X and Y axes) with a nanometer resolution were used. Distance between neighbor dimples was set to be 100 µm and accordingly the dimple density (D) was designed to be 20% as calculated from the equation for a square array of circular dimples:

(1) $$D\;=\text{ π}\;{\left(\frac{d}{2I}\right)}^{2}$$

where *d* is the diameter of a dimple and *I* is the distance between dimples. Such density was selected because in commercial self-solid-lubricated materials for plain bearings application typically fractions between 20% and 25% of the surface is actually covered by the solid lubricant agent inserts (e.g., graphite or MoS_2_) [37]. Dimple density was therefore designed so as to act similarly to such solid lubricant inserts, as it is assumed that the dimples can act as lubricant reservoirs, slowly releasing lubricant into the contact. The study of the influence of dimples geometry, density and array type are discussed in other works in literature [38,39] and was beyond the scope of this paper.

**Table 2:** Pulse energies of the ramped laser emission.

Pulse no.	Energy /mJ
1	0.12
2	0.23
3	0.14
4	0.11
5	0.08
6	0.06

It is worthwhile to note that no further treatment was applied for surface finishing or de-burring after laser processing. The morphology and geometrical parameters of the laser fabricated dimples and of the dimples pattern were estimated using the SEM. In particular, ten dimples per sample were analyzed to create a statistical estimation of the following geometrical attributes:

- dimple mean diameter: average diameter of each dimple was determined by measuring it in different positions across its centroid; the measurement line across dimple diameter was progressively rotated by 2 degree intervals around its center axis;
- dimple aspect ratio: the ratio between major axis and minor axis of the equivalent ellipse fitting the dimple profile.

On the other hand the dimple depth was estimated both by the difference of the focus positions at the dimple border and the bottom of the dimples with the SEM and by contact profilometry. For the latter measurement a Hommel Werke T1000 contact profilometer equipped with a 5 µm radius stylus was used. Moreover, Energy Dispersive Spectroscopy (EDS) analysis was performed on different zones of the generated dimples in order to verify whether the substrate was reached or not by the laser spot. For such analysis, an Oxford microprobe was used. Finally, some of the dimples were also mapped using the nanoindenter sensor (Hysistron UBI 950) as a scanning probe microscope (SPM).

### 2.3. Flat End Pin on Disc Sliding Wear Test

**Figure 1:**
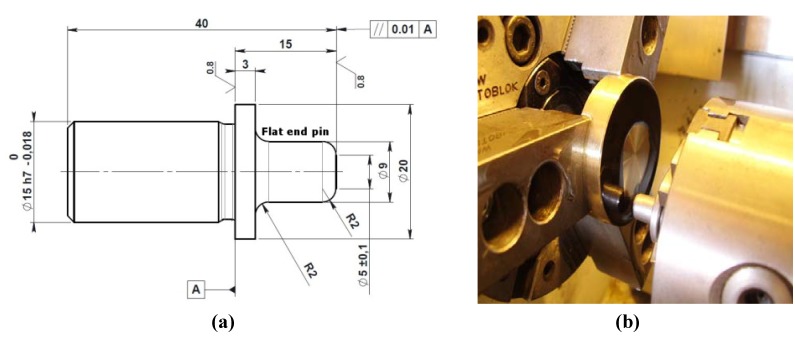
Details of (**a**) the counterpart flat end pin geometry and of (**b**) the flat end pin on disc assembly.

The wear tests were performed on plain and laser textured TiN and on WC/C coated 42CrMo4 disc samples. The tests on WC/C and plain TiN coated samples were performed to have reference data on traditional self-lubricating and hard coatings, not subjected to any post-processing. Tests were carried out with a flat end counterpart pin (Figure 1a), being its constitutive material the 18NiCrMo35 steel heat treated and carburized up to a superficial hardness of 58 HRC.

The nominal flat contact area is circular with a 5 mm diameter. The traditional pin on disc setup uses a rounded pin sliding on a flat sample. This generates a non-conformal contact, which gives a very high contact pressure, efficiently described by the classical Hertzian treatment. On the contrary, a conformal contact is here generated; in principle, such configurations are less aggressive in terms of contact pressure developed, but closer to the real sliding components operating mode. On the contrary, the extended nominal contact area favors the adhesion wear conditions. Further discussion on the actual contact pressure distribution is reported below. The test was carried out using a commercial lubricant oil, Castrol Hyspin AW 46. Such oil is generally used in sliders made from self-lubricating materials and has a viscosity index higher than 150. The test rig (Figure 1b) used for the wear test was a Cortini lathe specifically equipped with a load cell to monitor the tangential force. The total sliding distance of the test was 1000 m with periodical stops and inspections every 250 m. The lubricating oil was manually applied on the coated discs surface at the beginning of each 250 m test step. The working diameter calculated at the center of the sliding contact area was 35 mm and the rotational speed was 21 rpm. Two samples per coating configuration,* i.e.*, LST or plain TiN and WC/C, were tested.

**Figure 2:**
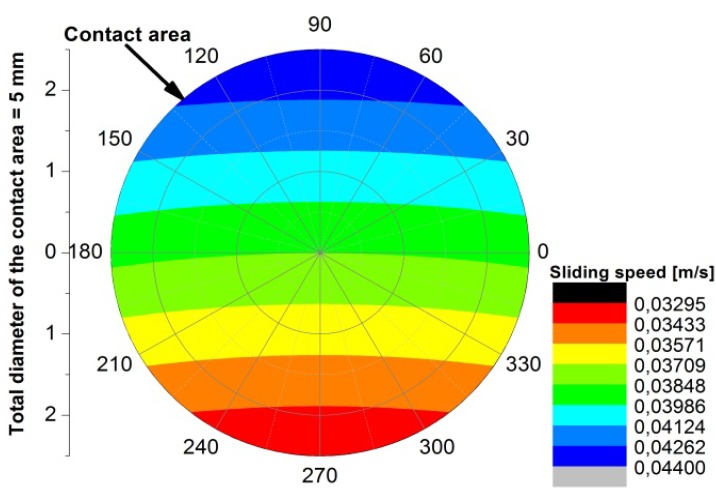
Map of the distribution of the sliding speed across the contact area.

**Figure 3:**
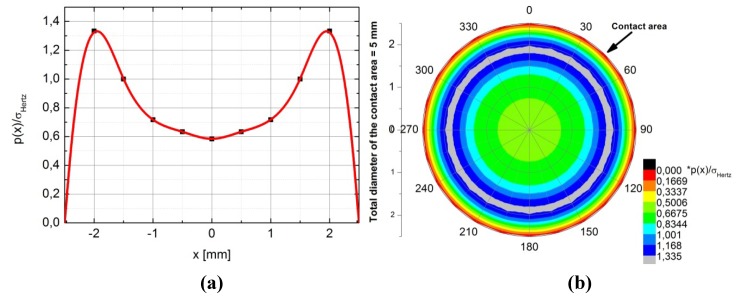
Distribution of the un-dimensional calculated parameter *p*(*x*)/σ_Hertz_ (**a**) along a diameter of the contact area and (**b**) across the whole contact area.

According to such parameters, the test was performed with an average sliding speed of 3.86 × 10^−2^ m/s and an applied normal force of 1760 N. Such level of force on the contact area provides a nominal pressure of about 90 MPa, giving a PV factor of 3.46 MPa × m/s. Actually, it is worthwhile to further discuss two aspects regarding the sliding speed and the contact pressure, which actually have both not uniform distribution across the contact area. As for the sliding speed, due to the flat pad on disc test configuration the sliding speed changes in the different points of the contact area, in particular the sliding speed increases as long as the distance from the center of rotation increases. Figure 2 shows a map of the local sliding speed applied over the contact area. As for the contact pressure, because the flat end pin and the disc are in conformal contact (non-Hertzian case), an important edge effect has to be considered. The calculation of static contact pressure distribution can be referred to the flat punch on a plane problem [40,41]. According to such treatment, the distribution of contact pressure along the diameter of the contact area has two symmetric peaks localized close to the edges of such area. Figure 3a shows the distribution of contact stresses as graphically derived by calculations reported in literature [42,43,44]. In such studies the distribution of the un-dimensional parameter *p*(*x*)/σ_Hertz_ along the diameter of the contact area is numerically calculated, being *p*(*x*) the calculated contact pressure and σ_Hertz_ the maximum contact pressure of the equivalent Hertzian case. Figure 3b shows a map of such parameter over the whole contact area.

Therefore, one can assume that within the same test different local PV conditions are applied over the contact area. In particular, in the region closer to the edges of such area and to the center of the disc rotation a combination of very high contact pressure and low sliding speed acts on the studied coated system. Such region will be frequently referred as the *internal portion of the wear track*. The combination of high contact pressure and slow sliding speed recalls quite well the operating conditions of the components such as sliders, cam on tappet system, *etc*., where potentially the surface modification system here studied could be applied.

At the end of each 250 m test step, the wear evolution was studied by estimating the average wear volume and the maximum wear depth. Both such values were achieved through profilometry measurements performed perpendicularly to the wear track with a stylus profiler, Hommel Werke T1000. The average wear volume is calculated by averaging the worn area measured at four different positions across the wear track and by multiplying it times the length of circumferential sliding path. From the same profiles recorded across the wear track also the maximum wear depth was monitored. This is also an important parameter since, as discussed below, it is able to put in evidence the presence of localized wear scratches. Finally, the friction coefficient was monitored during the entire test.

## 3. Results

### 3.1. Structural Characterization of Un-textured Coatings

The coatings thickness recorded by ball crater test on TiN and WC/C are 2.65 ± 0.13 µm and 2.17 ± 0.2 μm respectively. Recorded values of the reduced modulus (E), the nanohardness (H) and the maximum indentation depth (d_max_) for the TiN coating are respectively: E = 348.00 ± 17.15 GPa, H = 29.94 ± 2.60 GPa and d_max_ = 95.54 ± 4.72 nm. As for the WC/C coating such parameters are: E = 156.04 ± 11.70 GPa, H = 15.40 ± 1.76 GPa and d_max_ = 138.77 ± 8.93 nm.

**Figure 4:**
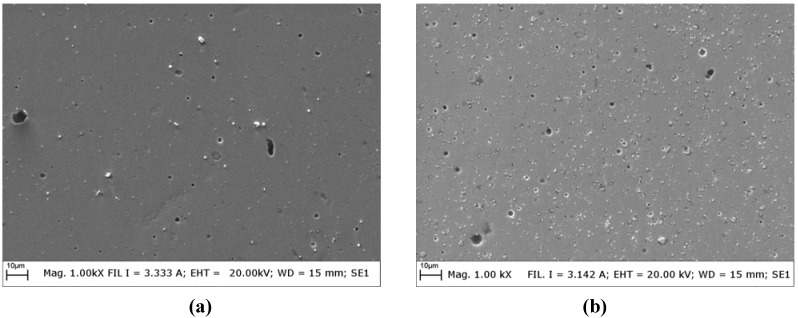
SEM images of (**a**) TiN and (**b**) WC/C coatings as deposited.

The as deposited morphology of the two coating grades (Figure 4) showed the presence of porosities and macro-particles, which are features typical of layers deposited by PVD process. On a qualitative level, WC/C exhibited a higher density of very fine macro-particles, whereas TiN presented indeed a lower density of macro-particles, but which have in turns larger average dimensions. In terms of porosity, the density of pores and their average dimensions are very similar for both coating grades.

### 3.2. Morphology of Textured Surface

**Figure 5:**
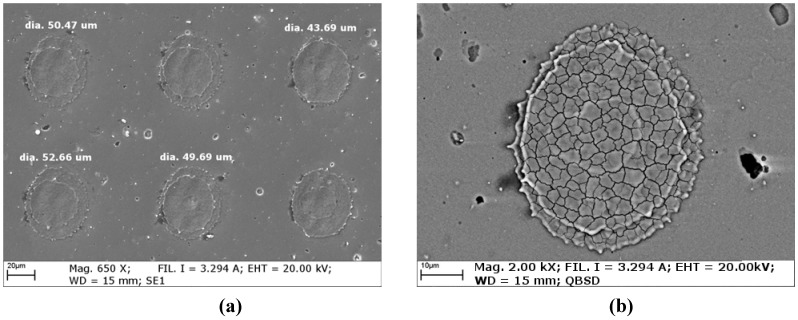
Morphology of laser treated TiN surface: (**a**) dimples array; (**b**) single dimple**.**

**Figure 6:**
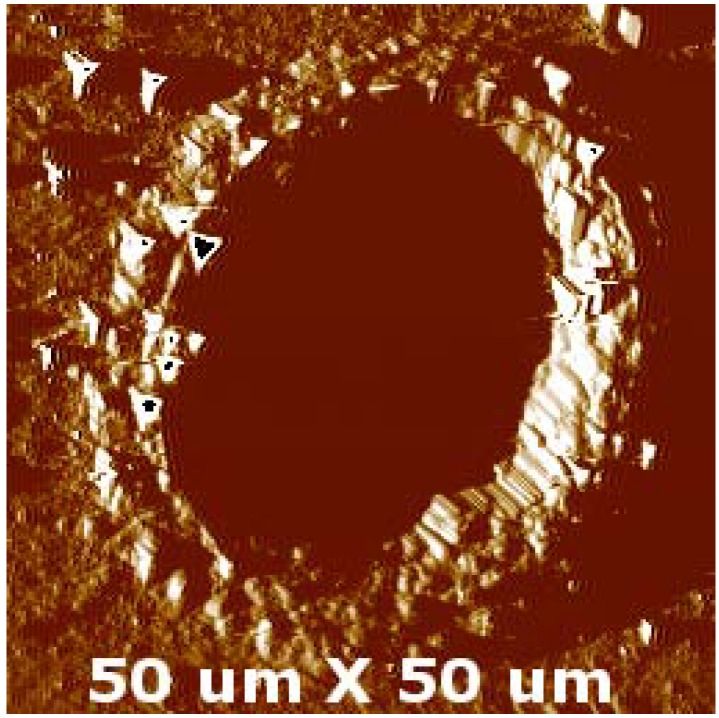
SPM image of a single dimple.

Figure 5 shows SEM images of the dimples generated on the coated disc surface. The analyses revealed that the texturing was consistent and repeatable over the whole treated surface. The dimple depth was estimated to be at maximum 2 µm, whereas the average diameter was measured as 48.7 ± 1.4 µm, as confirmed also by SPM images (Figure 6).

**Figure 7:**
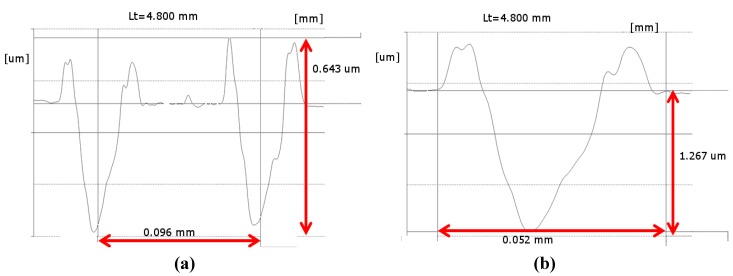
Recorded profiles of (**a**) two adjacent laser textured dimples and of (**b**) a single dimple.

Furthermore, using profilometry measurements, it was possible to distinguish the height of the burr at the edge, ranging between 0.4 to 0.7 µm (Figure 7a), and the actual depth of the dimple, ranging between 1.2 and 1.5 µm (Figure 7b). The spacing between two adjacent dimples was estimated to be *ca.* 100 µm in both the plane directions, forming a regular array of LST dimples. Moreover, dimples were substantially circular, since their average aspect ratio is 1.17 ± 0.02, and exhibited limited burring effect around their borders. It should be noted that ablation affected only the coating thickness and did not provoke any coating detachments, giving clues to the fact that a good adhesion level at the coating substrate interface can be maintained even upon laser texturing. Such an aspect was studied by the authors elsewhere [36]. The morphology of the dimples gives clear clues to the fact that limited melting of the coating occurred during the interaction between the laser spot and the coated sample. This resulted in the formation of thermal cracks within the laser affected areas and slightly outside of their edges, across the generated burr. Actually, such morphology is common in laser ablation with longer ns pulses [24,34], whereas in ps and fs laser ablation the ablated surfaces are characterized mainly by wrinkles and ripples [45].

### 3.3. Wear Behavior

**Figure 8:**
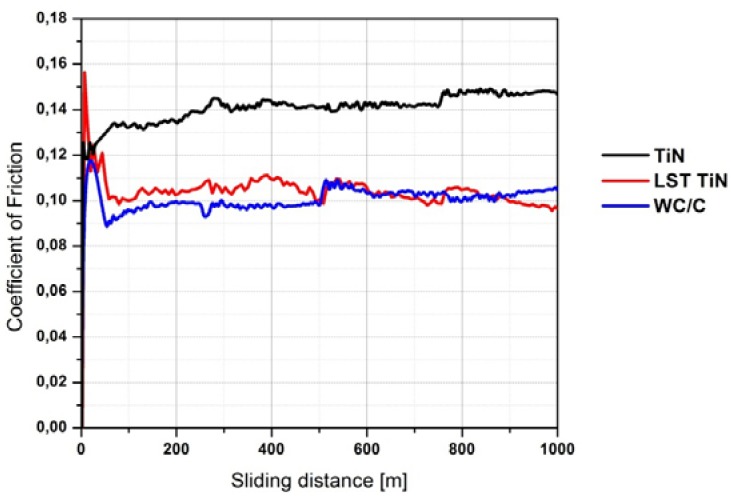
Friction coefficient versus sliding distance for the three coating grades.

In terms of coefficient of friction (COF) the three coating grades studied, plain TiN, LST-TiN and WC/C, presented different behaviors (Figure 8). The TiN COF exhibited a continuously increasing run-in stage, which rapidly stabilized at* ca.* 0.14. The WC/C and LST TiN exhibited both a sharp pronounced run-in peak followed by a steady state value of *ca.* 0.10. As for WC/C the initial peak is limited to 0.12, whereas for LST TiN reaches 0.16. For all the coating grades the steady state was achieved for sliding distances lower than 100 m. Generally, the run-in stage is due to the fact that at the beginning of the wear test the mating surfaces are modified by the contact actions and progressively match to each other, by adapting their initial roughness and morphology. In particular, in the WC/C system it is generally assumed that initially the coating misses part of its carbon content which is transferred onto the counterpart surface, giving the so-called graphitization effect which promotes a solid lubrication regime [46,47,48]. Later, after this stage, due to such mechanisms, the COF level is very low. On the contrary, the TiN coating has a very high hardness and therefore slightly changes its initial roughness, thus avoiding the formation of the COF peak. However, the surface asperities of such coating could act as abrasion pins on the counterpart surface. This could explain the observed progressive increase in COF, due to a higher abrasive contribution to friction. On the contrary, the high run-in peak exhibited by LST TiN can be due only to the modifications introduced by laser texturing. In particular, two can be the causes for the onset of such peak: (1) the local increase in roughness introduced by dimples; (2) the release of debris from laser fabricated dimples. The former source was due to the burrs provided along the edges of the dimples. A post polishing step applied just after the LST process, would limit this problem. As for the latter source, the LST process provided a diffused thermal cracking of dimples, as discussed before, and this indeed embrittles the coating, at least close to the dimples. Therefore, it can be expected that some portions of the coating is released as debris and is entrapped at the interface between the two mating bodies. At this stage, it is worthwhile to note that after such run-in the LST TiN assumed a COF level indeed lower than TiN and very similar to WC/C. Therefore, one can assume that the formation of debris, if present, is limited in amount and after the altered coating surface is smoothed by the run-in actions a more effective lubrication regime is achieved in LST TiN than in TiN. These aspects will be better addressed below when SEM images of the wear tracks are discussed.

**Figure 9:**
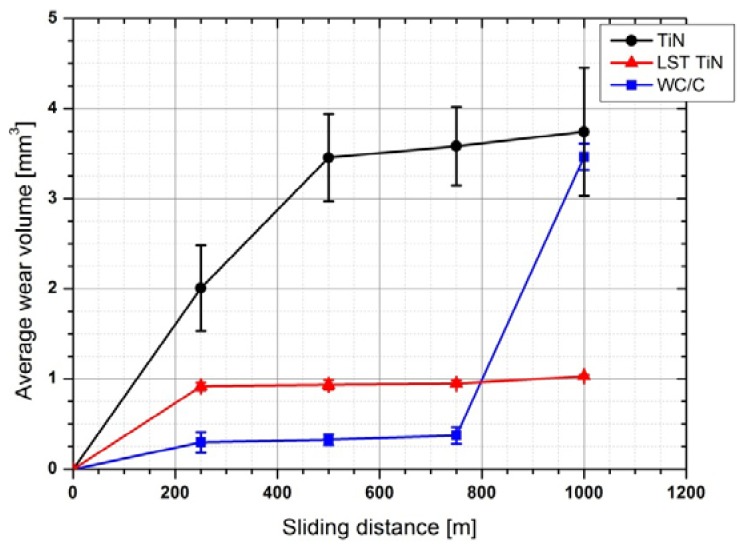
Average wear volume versus sliding distance.

**Figure 10:**
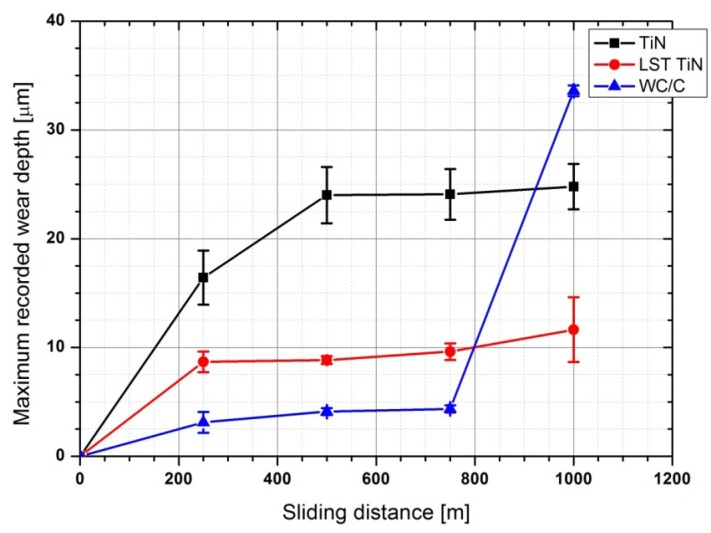
Maximum depth recorded within the wear track versus sliding distance.

In terms of wear, two parameters were mainly studied: (1) the average wear volume and (2) the maximum wear depth. The first parameter is calculated as described in Section 2.3 and can efficiently describe the average wear behavior of the three coating grades. The maximum wear depth is a more sensitive parameter in detecting localized damages introduced into the coated systems. Figure 9 and Figure 10 report the average wear volume and the recorded maximum wear depth as a function of sliding distance for the three coating grades.

From the point of view of wear volume, the TiN coating exhibited a very steep increase as the sliding distance was increased up to *ca.* 500 m. For higher sliding distances the wear volume continued to increase, but with a reduced slope. A real steady state of the wear process was not achieved by TiN, indicating a wear process, which even with low rates progresses through all the duration of the test. Furthermore, the wear level achieved by TiN at the end of the test was very high if compared to the other two coating grades studied. Actually, WC/C and LST TiN achieved wear steady state within the first 250 m (*i.e.*, after this initial stage they both exhibited very limited wear losses up to *ca.* 800 m of sliding distance). At up to 800 m of sliding distance, the wear levels achieved for both coatings are indeed lower than for TiN: one tenth for the WC/C and one fourth for the LST TiN. For higher sliding distance, the LST TiN continued to maintain a very low steady state wear behavior, whereas the WC/C presented a steep increase in wear volume, up to reaching roughly the same level achieved by TiN at the end of the test. This results in the fact that for long sliding distance LST TiN guaranteed a better resistance to wear in the test conditions applied. On the contrary, WC/C had effectively limited the wear effects up to 800 m of sliding distance, but after this it abruptly failed. Such sudden increase of wear rate can be explained only by the delamination of large portion of coatings, which provides an irregular contact between two mating surfaces.

The measurements of maximum wear depth substantially confirmed the general behaviors exhibited by the three coating grades in terms of wear volume. However, it has to be noted that apart from WC/C the other two coatings presented a maximum wear depth higher than their relative thickness just after 250 m. This means that deep localized wear scratches, able to remove the coating, were generated in the contact area of TiN and LST TiN very soon along the test duration. Probably, this occurred within the run-in stage. However, after 250 m of sliding distance the wear depth in LST TiN was stabilized at *ca.* 8 µm and maintained for the rest of the test. Whereas in TiN the wear depth continued to increase up to a level of *ca.* 25 microns at 500 m and then was stabilized at this level for the rest of the test. In LST TiN for sliding distance higher than 800 m, although the maximum wear depth exhibited a further slight increase the final value achieved, *ca.* 12 microns, is indeed very lower than for other coatings. As for the WC/C the wear depth slightly increased to less than 5 microns within the first 500 m, then maintained such levels up to 800 m and finally had a steep increase to achieve *ca.* 34 microns. This behavior agrees very well with that recorded in terms of the average wear volume, thus confirming that at this particular sliding distance a sudden deterioration of the coated surface occurred. In the case of maximum wear depth the level achieved by WC/C at the end of the test was even higher than that of TiN. Also according to such evaluation for long sliding distance the wear resistance of LST TiN resulted to be the best among the set of coating grades studied, although the tendency to increase at the end of the test reveals that a localized marked deterioration is starting to occur also in this coating.

**Figure 11:**
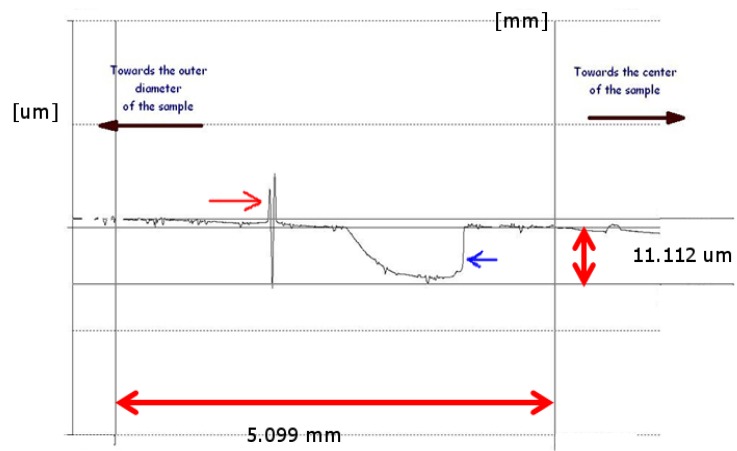
Recorded profile across the wear track on a LST sample after 1000 m of sliding distance.

**Figure 12:**
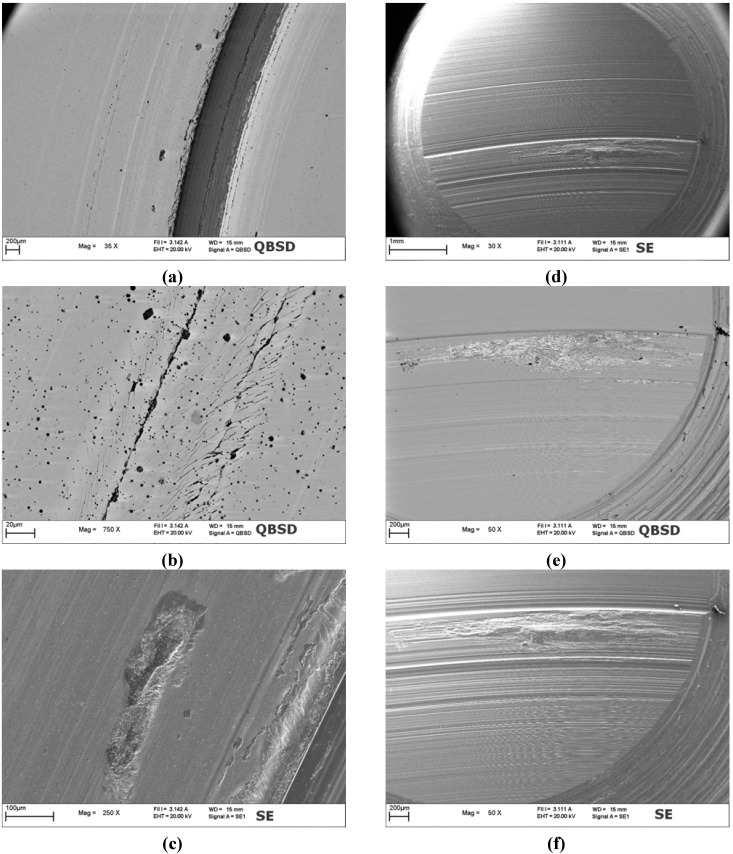
SEM micrographs of the WC/C coated disc and of the counterpart pin after 1000 m of sliding distance: (**a**), (**b**) and (**c**) large view and details of the wear track on coated disc; (**d**), (**e**) and (**f**) large view and details of the counterpart worn surface.

**Figure 13:**
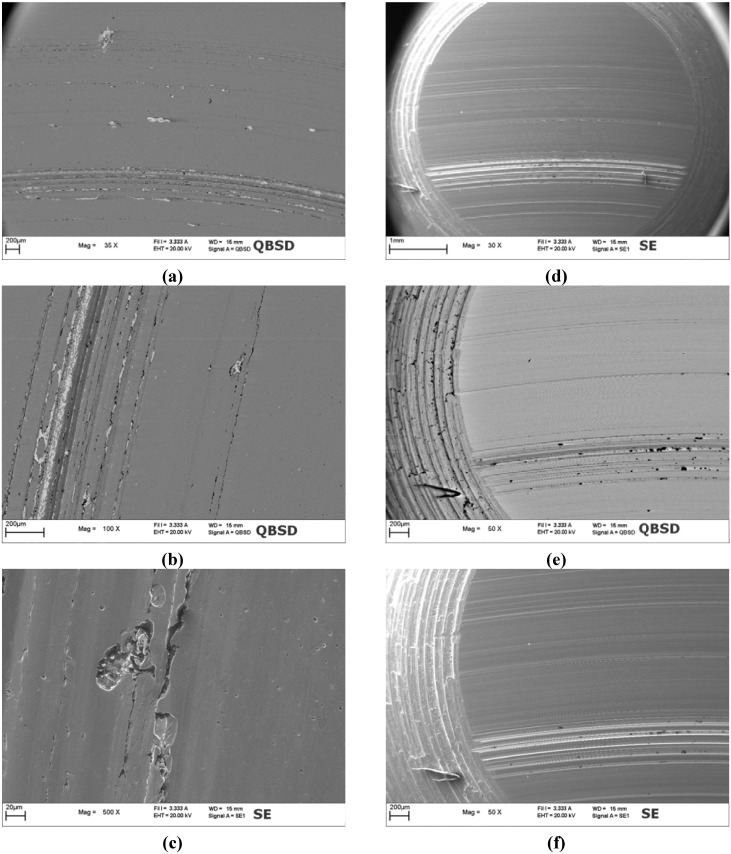
SEM micrographs of the TiN coated disc and of the counterpart pin after 1000 m of sliding distance: (**a**), (**b**) and (**c**) large view and details of the wear track on coated disc; (**d**), (**e**) and (**f**) large view and details of the counterpart worn surface.

**Figure 14:**
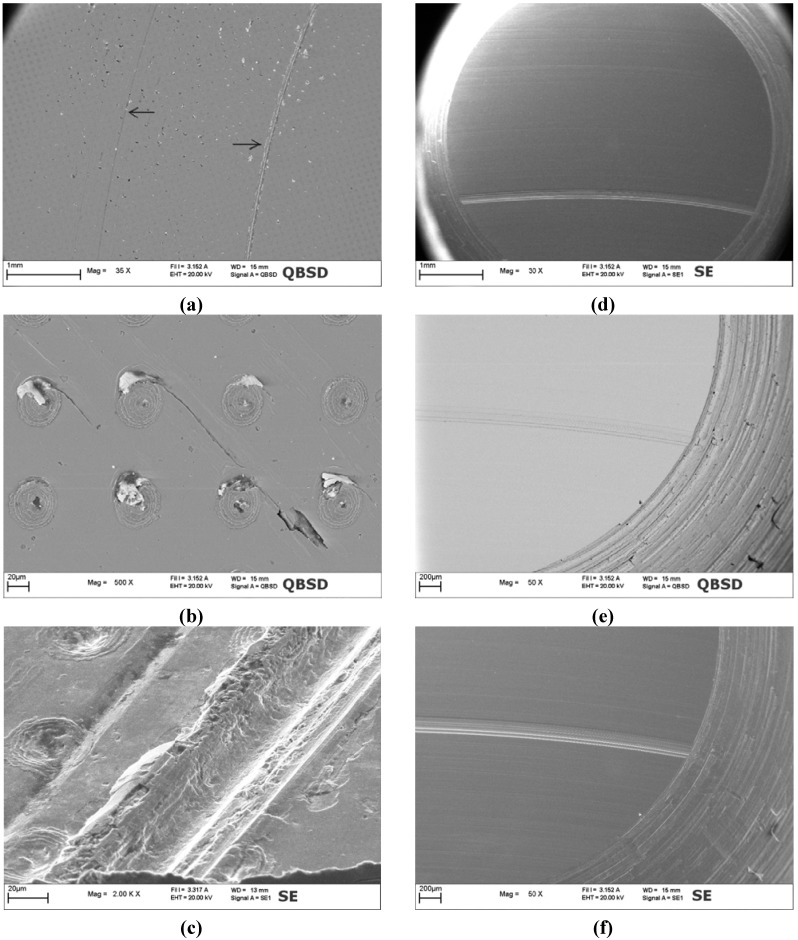
SEM micrographs of the LST TiN coated disc and of the counterpart pin after 1000m of sliding distance: (**a**), (**b**) and (**c**) large view and details of the wear track on coated disc; (**d**), (**e**) and (**f**) large view and details of the counterpart worn surface.

To better understand the analysis performed on average wear volume and maximum wear depth, further remarks have to be given concerning localization of some wear features across the wear track. Actually, for all the studied coating grades the material removal due to wear was not uniform across the wear track. This can be clearly observed in Figure 11, where a reference profile across the wear track on a LST TiN sample after 1000 m of sliding distance is reported. Actually, a large worn area (blue arrow) is visible in the internal portion of the wear track, *i.e.*, where the resulting sliding velocity was lower. On the contrary, in the external portion of the wear track, generally a mild wear occurred, but a localized very deep scratch (red arrow) close to the center of the contact area was also detected. Such features are common to all the studied coating grades, although different in intensity, *i.e.*, maximum recorded wear depth and the average wear volume as discussed before. The wear mechanisms were further studied through SEM analysis on the coated discs and of the relative counterpart pin after the wear test (Figure 12, Figure 13 and Figure 14).

Analysis of the WC/C coated disc showed a worn surface characterized by many different features. In the internal part of the wear track, a deep and wide portion of the coated system was removed (Figure 12a). In the rest of the wear track both deep scratches oriented in the sliding direction and localized detached pits could be detected (Figure 12b,c). From back scattered SEM images it was evident that within the wide portion of material removed and within the pits the coating was completely spalled off and the steel substrate was exposed. As for the deep scratches, it can be observed that in such locations, the coating was locally cut through its thickness and multiple cracks developed along their edges. Such cracks are oriented at *ca.* 45° with respect to the sliding direction far from the scratch and then tend to align to this direction closer to the scratch itself. This aspect of such cracks is very similar to that provided to coating by the scratch test actions, where a hard abrasive pin slides on the coated surface. One can assume that the deep cutting action is produced on WC/C by the abrasive action of wear debris entrapped into the contact. On the surface of the pin, which acted like the counterpart of WC/C, thin and wide scratches could be observed over all the contact area (Figure 12d). The presence of such scratches gives further clues to the fact that hard debris coming from coated disc were entrapped into the contact giving a third body abrasion effect. In the part of the pin surface that was in contact with the internal portion of the wear track also a massive material transfer could be detected (Figure 12e,f). The material transfer was also confirmed by EDS analysis. Such location corresponds to the region of the coated disc where a large portion of the material was removed, thus evidencing that a severe adhesive wear mechanism occurred on this portion of the coated surface.

The worn surface of plain TiN exhibited many deep scratches oriented in the sliding direction (Figure 13a,b) and some local pits (Figure 13c). Like for WC/C, also in the case of TiN the wear damages are more pronounced in the internal portion of the wear track. In particular, in such region many deep scratches cutting through the coating thickness were observed. In the rest of the wear track some large pits and shallow grooves were detected. Within shallow grooves, the coating was always present and the steel substrate was never exposed. On the contrary, in the deep scratches the coating was spalled off through a fragmentation mechanism similar to that observed in WC/C (Figure 13c). Such process resulted in the formation of many debris which generated a third body abrasive mechanism. However, the TiN is markedly harder than WC/C and this causes severe wear of both mating surfaces, leading to many deep scratches also on the counterpart pin (Figure 13d). Actually, instead of the intense material transfer occurred in WC/C the counterpart pin surface resulted to be severely worn with a minimum and localized build up (Figure 13e and f).

Finally, the LST TiN sample (Figure 14) exhibited a worn surface characterized by very few wear damages. In particular, only two pronounced scratches oriented along the sliding direction and located almost symmetrically with respect to the mid line of the wear track were detected (black arrows in Figure 14a). The symmetrical position of these two scratches can be probably related to the symmetric locations of the two contact pressure peaks (see Figure 3a and the related discussion at Section 2.3). The scratch lying in the internal portion of the wear track is deeper and wider. This closely recalls what happened in the other two coatings studied, *i.e.* that this portion of the sliding contact area was subjected to more severe wear conditions. Apart from these two main scratches, on the rest of the wear track only shallow grooves and some damages to the dimples were detected (Figure 14b).

Actually, laser textured dimples are sometime partially damaged, *i.e.*, the main part of the dimple is still present and unaltered, but on the edge which firstly enters in contact with the counterpart pin the burr was fractured thus forming hard debris. At these sites part of the laser altered coating was removed creating a crater, where steel debris loosen by the counterpart pin were entrapped (lighter particles in the back scattered SEM image in Figure 14b). Starting from these damaged regions of the dimples sometime also channeling cracks nucleated and propagated towards the neighboring dimples following the sliding direction. This process of course embrittled the LST TiN coating and further produced hard debris, which could be entrapped into the sliding contact area. In the region where the contact pressure were higher this ultimately resulted in the complete removal of the coating and the plowing of the steel substrate (Figure 14c). The coating failure was observed to proceed through a fragmentation process very similar to that observed on plain TiN surface. On the counterpart pin of the LST TiN coated sample very shallow polishing grooves and only a localized deep wear scratch could be detected (Figure 14d). As usually such a pronounced groove was localized in the part of the pin which was in contact with the internal portion of the wear track of the coated sample. In such locations, deep plowing of the carburized steel pin was observed, but the material transfer from the coated surface was completely absent (Figure 14e,f).

## 4. Discussion

The wear mechanisms sequence was at least in the early stage similar for WC/C and TiN coating grades studied. In the first stage of the wear test, the predominant wear mechanism was adhesion. In the internal portion of the wear track, where the sliding velocity regime is lower, the most severe adhesive wear action occurred. This confirms that although lubricated contact was applied the high pressure and low velocity conditions resulted in boundary lubrication regime. Therefore, the peaks of the asperities of two mating surfaces got in contact, thus resulting in adhesion. This is basically confirmed by the portions of the coated surface, which are detached (pits) and by the material build up onto the counterpart pin. In a second stage, the debris detached from coated surfaces were entrapped at the contact interface between the two bodies and provided third body abrasive wear mechanisms. In TiN coating such debris are very hard, sharp and coarse, thus easily resulting in the formation of wedges on the carburized steel pin and cutting on the hard coating. At the end of the test the internal portion of the wear track and the counterpart region of the carburized steel pin was almost completely covered by deep scratches. In addition, in the rest of contact area, heavy damages were detected on the coating. Actually, considering also the shape of the average wear volume or maximum depth versus sliding distance curves one can conclude that very early during the test many large debris were released into the contact region from the TiN coated sample. Since TiN is much harder than WC/C, the third body abrasive wear mechanism was thus severe and provided a steep wear rate. In a later stage of the test, the amount of released debris did not markedly increased and the entrapped debris started to round their edges and to be less effective in abrasion. Therefore, a decrease in the wear rate occurred. This would explain also the two stages shape of the COF curve.

As for the WC/C, it is a self-lubricating coating; *i.e.* it is a material which works by the progressive material consumption (mainly the carbon phase) so as to generate a solid lubricant film interposed between the two mating surface. However, in the specific wear test here described WC/C worked properly until *ca.* 800 m, giving up to such distance both a very low volume and maximum wear depth. Later on, an intense material loss occurred from the coated surface and transferred onto the counterpart surface. The extension of such phenomenon is so high that it can be referred as galling of the mating surfaces. This occurred suddenly and caused also an intense plastic deformation of the exposed substrate, which was rapidly damaged by the wear action. Like in TiN wear debris released into the contact region provided scratches. However, since the hard phases in such a coating are fine small WC grains, the abrasive action is less severe than in TiN.

The behavior of LST TiN is very interesting. Actually, TiN coating has a very high hardness, but it was clearly demonstrated that in heavy loaded and low sliding speed condition suffered by adhesive wear followed by coating fragmentation due to the abrasion from hard debris. The dimples pattern textured on TiN markedly reduced the tendency to adhesive wear for the two mating surfaces. This is clearly demonstrated by the COF level achieved, which was lower than plain TiN (very similar to WC/C) and could be maintained stable for long sliding distance. Furthermore, the very limited amount of detachments and scratches revealed both on the coated surface and on the counterpart pin confirmed this. In addition, the maximum recorded wear depth and the average wear volume were indeed lower than the other two coating grades. It can be even supposed that the limited coating debris release actually occurred in the very first stage of the wear test, *i.e.*, during the run-in. As already discussed, for such coating the pronounced run-in effects can be related to the increase in roughness introduced by LST process and to the presence of burrs and ridges on the edges of the dimples. This indicates that by enhancing the quality of dimples edges, even better results could be achieved. Such improvement can be obtained either by further optimizing the LST process or by introducing a post polishing treatment.

The enhancements introduced by laser surface texturing of hard TiN coatings can be ascribed to different mechanisms. First the dimpled surface generates a discontinuous contact with the counterpart pin, thus giving a real contact area which is indeed lower than the nominal one.. This initially favors the reduction of the adhesion force, which is proportional to the contact area, but afterwards results in a concentration of the bearing loads in localized areas which are more easily subjected to plastic strains providing on the contrary an increase in the real contact area, thus limiting the previous beneficial effect. Second the dimples are superficial cavities were the oil used to lubricate the contact can be more effectively maintained and slowly released into contact. This aspect could be even favored by the fact that the thermal cracks generated within the dimples act as a sort of artificial porosities and more efficiently absorb the lubricant. Third, the dimples can entrap the wear debris introduced into the contact interface, thus reducing the third body effect. Fourth as reported by literature [3,4,5,6,10,12,14], such surface dimples can also promote a dispersed micro elastic-hydrodynamic lubrication regime that may help to maintain the two mating surfaces separated, even at low sliding speed. The improvements here reported in terms of wear are considered to be ascribed mainly to the second and third effects. The effectiveness of the debris trapping effect was argued by observing the presence of several wear debris into the dimples at the end of the wear test. A further confirmation of this was achieved by the fact that also the LST TiN counterpart pin exhibited less abrasion scratches if compared to the pins used as counterpart for plain TiN and WC/C. Clues for the effectiveness of the better ability to lubricate mating surfaces can be provided by the better behavior of LST TiN in terms of friction coefficient and by the reduced tendency to have material transfer (adhesive wear) if compared to plain TiN.

## 5. Conclusions

An effective laser texturing processing of hard thin coating was achieved using fiber laser. The width to depth ratio of the generated dimples was efficiently controlled. Once laser processing was optimized, a dimple pattern with specific geometrical features and density was applied on TiN coatings so as to obtain a 20% coverage area. Sliding wear tests were performed with flat on flat mating surfaces configuration applying very high PV factor. Lubrication with oil was applied manually and discontinuously (every 250 m) and this provided boundary lubrication conditions. Reference tests on plain TiN and a self-lubricated coating (WC/C) were also performed.

The major result of this study is that it was demonstrated that in heavy loaded conditions, low sliding speed, boundary lubricated conditions and with conformal contact (*i.e.* flat pad on a flat plane), the introduction of LST on hard thin TiN coating can increase the wear resistance by *ca.* 70% in terms of wear volume and by *ca.* 45% in terms of maximum wear depth. This was achieved also along with a reduction of the coefficient of friction, if compared to plain TiN. Actually, LST TiN recorded a stable COF, which was very similar to that of a self-lubricating coating, WC/C, but guaranteeing much more wear resistance. The beneficial effects of LST on TiN are due to a combination of many factors: capacity to entrap wear debris, modification of the contact area and ability to achieve a more efficient lubrication.

It is worthwhile to note that at the end of the test, few wear scratches are present in the LST TiN coatings and locally in such regions the coating was completely removed and the steel substrate was also subjected to abrasive wear. Nevertheless, such severe wear effects are very localized and limited in amount across the wear track.

Another important result is that the LST was obtained using a fiber laser source with ns pulse frequency, which is therefore less costly than the more complicated ps and fs sources. Actually, thermal cracking was introduced within the fabricated dimple, but this did not provoke a marked fragmentation of the coating even upon severe wear conditions. Wear cracks were actually generated by the sliding action, but they were mainly due to fragmentation of burrs and ridges and they rarely connected neighboring dimples. Therefore, it could be expected that further increase in the wear resistance of LST coatings can be obtained by reducing such cracking effect, for example using a pre-heating of the substrate during LST operations.
